# Clinical Decision‐Making in Peptic Esophageal Stenosis: To PEG or Not to PEG?

**DOI:** 10.1002/ccr3.73381

**Published:** 2026-08-21

**Authors:** Junus Benjamin Cokovic, Christian Prinz, Leonard Fehring

**Affiliations:** ^1^ Faculty of Health Witten/Herdecke University Witten Germany; ^2^ Clinic for Gastroenterology, Hepatology, Endocrinology and Diabetology Helios University Hospital Wuppertal Wuppertal Germany

**Keywords:** decision‐making, dilatation, esophageal squamous cell carcinoma, esophageal stenosis, esophagectomy, gastrostomy

## Abstract

Esophageal strictures can cause dysphagia and malnutrition requiring enteral feeding. We report a 78‐year‐old woman with therapy‐refractory distal esophageal stenosis initially considered benign. PEG tube placement for nutritional support revealed an unexpected squamous cell carcinoma. The patient underwent esophagectomy. We highlight diagnostic challenges and the need for careful PEG tube indication.

## Key Clinical Message

Nutritional management of treatment‐refractory esophageal stricture should consider malignancy risk, resection likelihood, and nutritional urgency. PEG tube supports long‐term nutrition in benign stenosis but remains controversial due to concerns regarding gastric conduit formation, postoperative complications, and tumor seeding. Following interdisciplinary assessment, PEG tube (preferably via push method) or alternatives (PEJ tube, parenteral nutrition) may be appropriate.

## 1 Introduction

Esophageal strictures, defined as pathological luminal narrowing of the esophagus, represent a relatively uncommon condition with increasing incidence in older populations [1]. Clinically, they manifest predominantly as progressive dysphagia to solid and/or liquid intake and are frequently accompanied by non‐cardiac chest pain [2]. In primary care settings, the majority of cases are peptic in origin, arising as a complication of gastroesophageal reflux disease (GERD). GERD is the most prevalent gastrointestinal disorder worldwide, affecting approximately 20% of individuals in Western countries, and is mainly driven by lifestyle factors, such as alcohol consumption, smoking, and weight gain [3]. While short‐term mucosal irritation is often self‐limiting, persistent or severe injury to the esophageal epithelium leads to significant scarring, ultimately resulting in stenosis [4]. Furthermore, sustained epithelial damage and cellular stress increase the risk of malignant transformation [5].

In patients with esophageal stenosis, adequate oral nutritional intake is frequently compromised due to mechanical obstruction and progressive dysphagia. In such cases, placing a percutaneous endoscopic gastrostomy (PEG) tube often represents the most effective method to bypass the stenotic segment and ensure sufficient enteral nutrition. During this procedure, a large‐bore plastic catheter is inserted transdermally into the stomach under endoscopic guidance, allowing for direct access to the gastrointestinal lumen [6]. The PEG tube is specifically intended for long‐term enteral nutrition. Current clinical guidelines recommend that PEG tube placement be considered when enteral feeding is expected to be necessary for at least 2–3 weeks [7]. Prospective clinical studies have shown that in most cases, supplemental nutrition via PEG tube can prevent progressive weight loss and thus maintain nutritional status, although complete recovery is rare even in benign conditions [8].

## 2 Case History

A 78‐year‐old woman was referred to our surgical department for functional staging prior to potential operative management of a long‐segment esophageal stricture. The stenosis had initially been classified as benign and peptic in origin and has been repeatedly treated by endoscopic bougienage at an external institution without sustained clinical success. At presentation, the patient reported progressive dysphagia, restricted to liquid intake, and marked involuntary weight loss of approximately 30 kg over the preceding two years.

Her medical history was further complicated by intra‐ and extrahepatic cholestasis secondary to choledocholithiasis, for which a common hepatic duct stent had been placed previously. Definitive biliary sanitation via endoscopic retrograde cholangiopancreatography (ERCP) has not been achieved due to the inability to traverse the esophageal stenosis endoscopically. Initial preoperative risk assessment revealed severe obstructive pulmonary disease (FEV_1_ at 0.87 L), rendering the patient unsuitable for immediate surgical intervention. The patient was therefore transferred to our gastroenterological department for conservative therapy.

## 3 Investigations and Treatments

**FIGURE 1:**
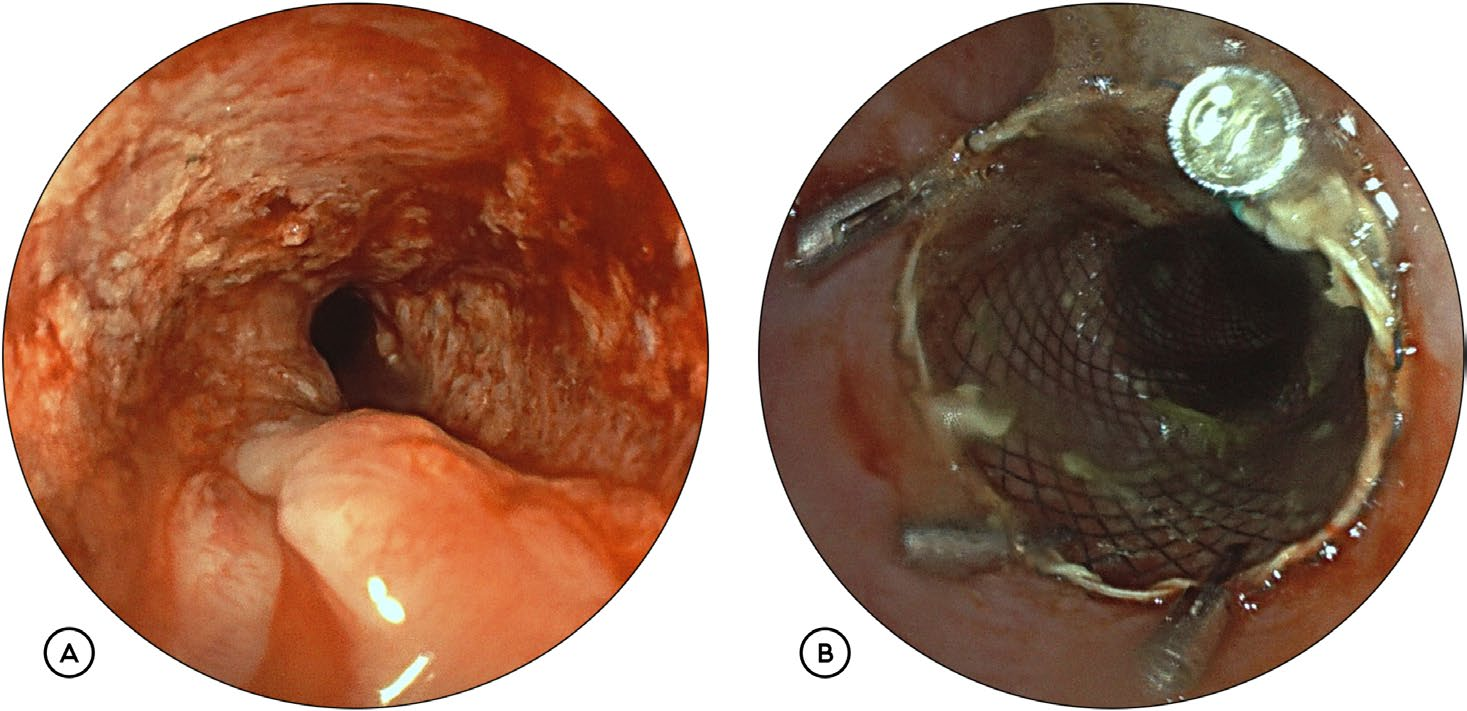
(A) Severe reflux esophagitis with ulcerations (LA‐D). (B) Stent fixation with four clips.

During the following weeks, the patient required recurrent hospital admissions due to progressive dysphagia, intermittent bolus impaction, and ongoing weight loss. Endoscopic evaluation consistently demonstrated a high‐grade distal esophageal stricture (31–36 cm from the incisors) with marked inflammatory changes and granulation tissue (see Figure 1A). A CT scan confirmed these findings without evidence of malignancy at that time. Despite multiple endoscopic interventions, including Savary bougienages and balloon dilatations (9–15 mm), as well as implantation and repositioning of a fully covered esophageal stent with additional clip fixation (see Figure 1B), the clinical course remained refractory. The disease trajectory was further complicated by proton pump inhibitor (PPI)‐resistant reflux esophagitis, repeated stent migration, partial stent embedding resembling a buried bumper phenomenon, and secondary Candida esophagitis. Pharmacological therapy included high‐dose PPIs, combined PPI and H_2_‐receptor antagonist therapy, and short‐term systemic antifungal treatment with fluconazole.

**FIGURE 2:**
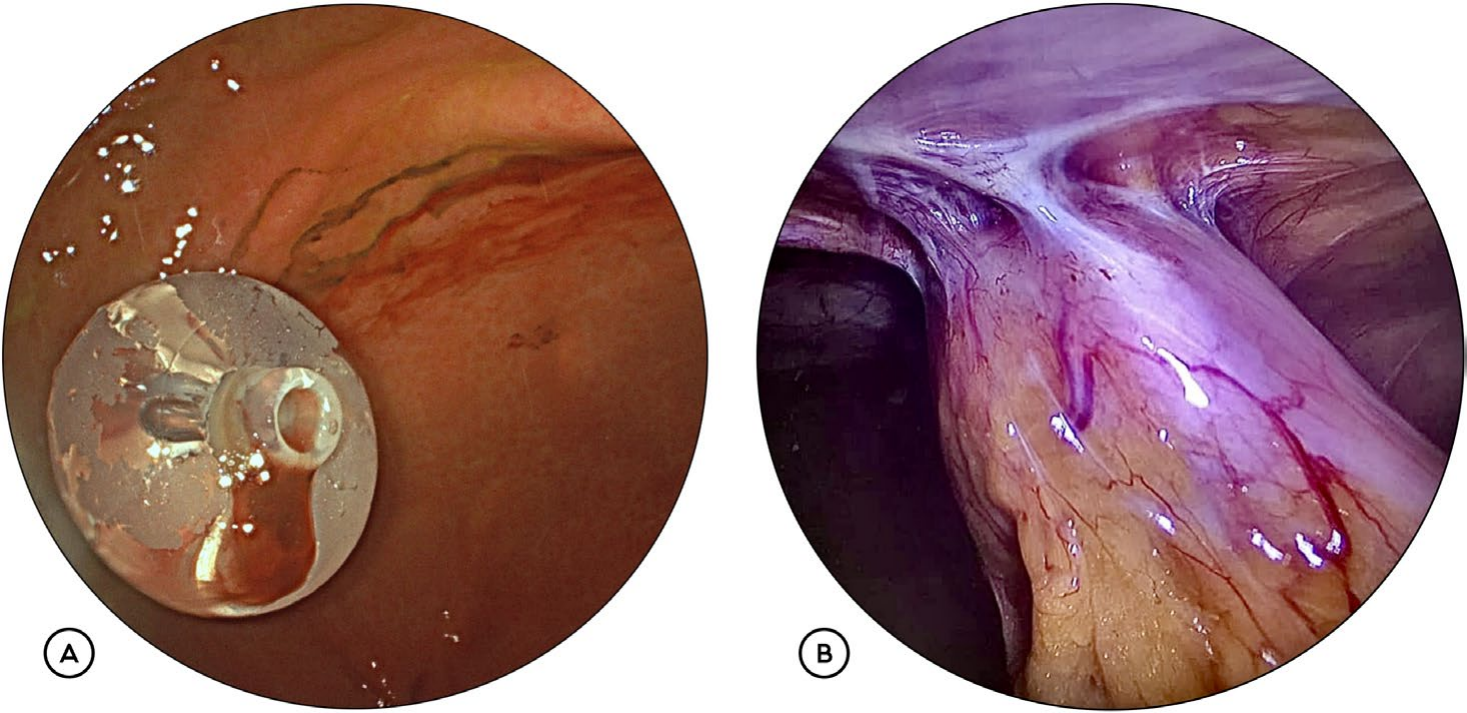
(A) Endoscopic view of PEG tube. (B) Laparoscopic view of PEG tube.

Due to progressive malnutrition and a cumulative weight loss of nearly 40 kg, a PEG tube was placed after exclusion of surrounding malignancy by targeted biopsies (see Figure 2A). During this procedure, repeated biopsies from the esophageal stricture unexpectedly revealed a moderately differentiated, keratinizing invasive squamous cell carcinoma.

Subsequent staging by contrast‐enhanced CT of the chest and abdomen showed marked esophageal wall thickening but no distant metastases. Following interdisciplinary tumor board discussion, surgical treatment was recommended. The patient was transferred back to the surgical department and underwent a hybrid Ivor Lewis esophagectomy consisting of laparoscopic gastric mobilization and transthoracic en bloc esophagectomy with two‐field lymphadenectomy, combined with simultaneous cholecystectomy (due to severe cholestasis).

## 4 Outcome and Results

Postoperative recovery was uneventful, and the patient was discharged on postoperative day 13.

Final histopathology confirmed a moderately differentiated squamous cell carcinoma staged as pT3 pN2 (5/33) R0 (stage III B). A structured oncological follow‐up program was initiated.

## 5 Discussion

Patients with esophageal strictures often develop significant weight loss, primarily due to mechanical impairment of bolus transit [9], with the extent of dysphagia being associated with the nutritional deficit [10]. In the present case, a cumulative weight loss of nearly 40 kg necessitated urgent nutritional stabilization. This was of central importance both independently of the underlying disease and with regard to any potential subsequent surgical treatment. At the time the indication was established, the condition was still considered benign, which is why the decision was made to place a PEG tube. This case particularly illustrates the diagnostic challenge of differentiating benign peptic lesions from occult malignancy in the context of severe esophageal stenosis accompanied by extensive inflammation, which additionally complicated representative tissue sampling. Repeated histopathological analyses consistently demonstrated severe chronic inflammation and granulation tissue without evidence of dysplasia or malignancy. In this context, it should be considered that biopsies obtained during esophagogastroduodenoscopy (EGD) are superficial and do not allow conclusions regarding possible malignant cells in the deeper layers of the tissue. In retrospect, the combination of progressive dysphagia, marked weight loss, and refractoriness to therapy represented important clinical red flags that should have sustained a high index of suspicion for an underlying malignancy despite initially negative histopathological findings. Furthermore, the diagnostic workup might have benefited from endoscopic ultrasound evaluation to assess mural invasion and locoregional lymphadenopathy. However, this technique is frequently technically unfeasible in cases of high‐grade stenosis.

Had a malignant origin been suspected at that time, an alternative form of nutritional support, such as percutaneous endoscopic jejunostomy (PEJ) or parenteral nutrition, would have been preferentially considered [11]. However, these approaches are also associated with specific limitations and challenges. Placement of a PEJ tube, for instance, may initially appear to be a suitable alternative, but the procedure is technically more demanding, and its success depends on the physician's expertise [12]. Parenteral nutrition, on the other hand, can also serve as a temporary bridging strategy in patients in whom enteral access is not possible or where diagnostic clarification is still pending. Nevertheless, its long‐term use is limited by the risk of infectious and metabolic complications [13]. Regardless of the method, the timely restoration of adequate nutritional status was the primary focus, as this is an essential prerequisite for further therapeutic planning and also, concerning the later surgery, for reducing postoperative complications, shortening the recovery phase, and improving overall surgical resilience [14]. The importance of nutritional support is also stated in the guideline: Patients with high metabolic risk should receive nutritional therapy before surgery, even if the surgery must be postponed [15].

PEG tube placement is generally considered a relative contraindication in patients with suspected or confirmed malignancy requiring esophagectomy with gastric pull‐up, due to potential implications for subsequent reconstruction [16]. However, several studies have demonstrated that a preexisting PEG tube does not adversely affect Ivor Lewis esophagectomy, and gastric conduit formation can usually be performed without relevant limitations or increased postoperative morbidity [17, 18, 19, 20]. Nevertheless, PEG‐related fibrosis or scarring of the anterior gastric wall may theoretically impair gastric conduit preparation and perfusion, particularly when the PEG is placed at the caudal stomach near the right gastroepiploic vessels (see Figure 2B). As these vessels represent the primary vascular supply for reconstruction, injury or compromise may result in impaired gastric perfusion [21, 22]. Adequate perfusion is critical, as hypoperfusion increases the risk of serious complications such as ischemia, anastomotic leakage, and gastric conduit necrosis [23]. In severe cases, alternative reconstruction using jejunal or colonic interposition may be required. To minimize those risks, the alternative nutritional strategies mentioned above could be considered, particularly if an esophageal resection is anticipated.

Another relevant aspect concerns the oncological implications of PEG tube placement in cases of undiagnosed malignancy. Though rare, tumor seeding along the gastrostomy tract has been described in some case reports and should therefore be taken into account before the intervention [24, 25, 26]. To minimize the spread of tumor cells by avoiding passage through the cancerous area, the PEG tube placement should be performed in direct percutaneous introducer technique (push method) [27, 28], as it was done in our case. A meta‐analysis by Siu et al. demonstrated that this approach significantly reduced the event rate of gastrostomy site metastasis (0.56% with pull method vs. 0.29% with push method) [29]. However, due to concerns regarding data quality, as well as reports describing tumor cell dissemination via hematogenous spread even with the push method, the American Society for Gastrointestinal Endoscopy (ASGE) refrained from exclusively recommending the push over the pull method in its latest guideline. While advisable, it should not be mandatory. Furthermore, higher‐quality studies on implantation metastasis will be necessary to more accurately assess the risk [30].

## Author Contributions

**Junus Benjamin Cokovic:** visualization, writing – original draft, writing – review and editing. **Christian Prinz:** supervision, writing – review and editing. **Leonard Fehring:** project administration, supervision, writing – review and editing.

## Funding

The authors have nothing to report.

## Consent

The patient provided written informed consent for the presentation and publication of the clinical history described in this case report.

## Conflicts of Interest

The authors declare no conflicts of interest.

## Data Availability

The authors have nothing to report.
